# 2-Bromo-4-chloro-6-[(*E*)-(2-chloro­phen­yl)imino­meth­yl]phenol

**DOI:** 10.1107/S1600536809007181

**Published:** 2009-03-06

**Authors:** Xinli Zhang

**Affiliations:** a Department of Chemistry, Baoji University of Arts and Science, Baoji, Shaanxi 721007, People’s Republic of China

## Abstract

The title compound, C_13_H_8_BrCl_2_NO, was obtained by reaction of 3-bromo-5-chloro­salicylaldehyde and 2-chloro­benzenamine in methanol. The mol­ecule displays an *E* configuration with respect to the imine C=N double bond. The dihedral angle between the two benzene rings is 4.57 (11)°. The mol­ecular conformation is stabilized by an intra­molecular O—H⋯N hydrogen bond. In the crystal structure, mol­ecules are linked by inter­molecular C—H⋯O hydrogen-bonding inter­actions into zigzag chains running parallel to the *b* axis. Inter­molecular Br⋯Cl [3.5289 (11) Å] and Cl⋯Cl [3.5042 (12) Å] inter­actions are present.

## Related literature

For the biological activities of Schiff base complexes, see: Cukurovali *et al.* (2002 ▶); Tarafder *et al.* (2002 ▶); Ali *et al.* (2002 ▶). For halogen–halogen inter­actions, see: Saruma *et al.* (1986 ▶); Moorthy *et al.* (2002 ▶).
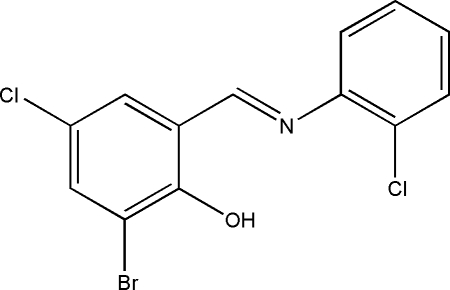


## Experimental

### 

#### Crystal data

C_13_H_8_BrCl_2_NO
*M* *_r_* = 345.01
Monoclinic, 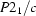

*a* = 8.4299 (10) Å
*b* = 14.0115 (16) Å
*c* = 11.4194 (14) Å
β = 104.5120 (10)°
*V* = 1305.8 (3) Å^3^
*Z* = 4
Mo *K*α radiation
μ = 3.54 mm^−1^
*T* = 298 K
0.45 × 0.38 × 0.36 mm

#### Data collection

Siemens SMART CCD area-detector diffractometer
Absorption correction: multi-scan (*SADABS*; Siemens, 1996 ▶) *T*_min_ = 0.230, *T*_max_ = 0.279
6450 measured reflections
2295 independent reflections
1726 reflections with *I* > 2σ(*I*)
*R*_int_ = 0.038

#### Refinement

*R*[*F* ^2^ > 2σ(*F* ^2^)] = 0.032
*wR*(*F* ^2^) = 0.077
*S* = 1.03
2295 reflections
164 parameters
H-atom parameters constrained
Δρ_max_ = 0.39 e Å^−3^
Δρ_min_ = −0.58 e Å^−3^

### 

Data collection: *SMART* (Siemens, 1996 ▶); cell refinement: *SAINT* (Siemens, 1996 ▶); data reduction: *SAINT*; program(s) used to solve structure: *SHELXS97* (Sheldrick, 2008 ▶); program(s) used to refine structure: *SHELXL97* (Sheldrick, 2008 ▶); molecular graphics: *SHELXTL* (Sheldrick, 2008 ▶); software used to prepare material for publication: *SHELXTL*.

## Supplementary Material

Crystal structure: contains datablocks I, global. DOI: 10.1107/S1600536809007181/rz2298sup1.cif

Structure factors: contains datablocks I. DOI: 10.1107/S1600536809007181/rz2298Isup2.hkl

Additional supplementary materials:  crystallographic information; 3D view; checkCIF report

## Figures and Tables

**Table 1:** Hydrogen-bond geometry (Å, °)

*D*—H⋯*A*	*D*—H	H⋯*A*	*D*⋯*A*	*D*—H⋯*A*
O1—H1⋯N1	0.82	1.86	2.586 (3)	147
C11—H11⋯O1^i^	0.93	2.56	3.324 (5)	139

Symmetry code: (i) 
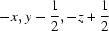
.

**Fig. 1.**
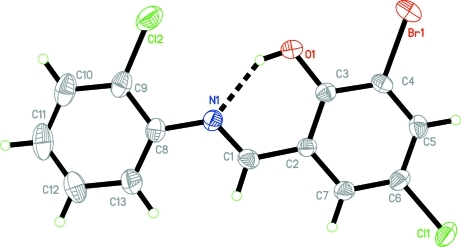
The structure of the title compound with 30% probability ellipsoids. H atoms are shown as spheres of arbitrary radii. The dashed line represents a hydrogen bond.

**Fig. 2.**
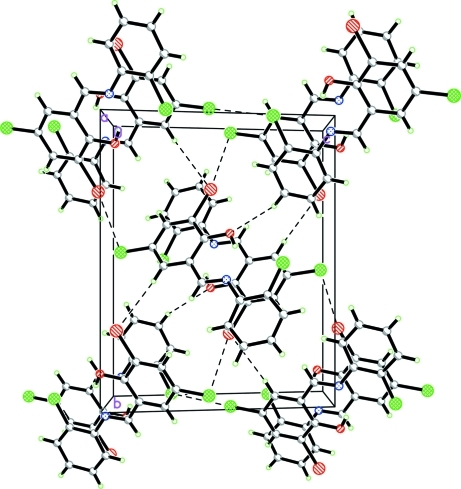
The crystal packing of the title compound viewed along the a axis. Halogen-halogen interactions are shown as dashed lines.

## supplementary crystallographic information

### Comment

Schiff base complexes are of great interests for inorganic and bioinorganic chemistry. To the best of our knowledge, in the past two decades Schiff base ligands have demonstrated significant biological activities and new examples have been tested for their antitumor, antimicrobial and antiviral activities (Tarafder *et al.*, 2002; Cukurovali *et al.*, 2002; Ali *et al.*, 2002). As an extension of the work on the structural characterization of Schiff base compounds, the crystal structure of the title compound is reported here.

The molecular structure and crystal packing of the title compound are illustrated in Fig. 1 and 2, respectively. The C1═N1 bond distance (1.279 (4) Å) is shorter than expected. The molecule is not strictly planar, the maximum deviations from the planarity are 0.199 (5) and 0.162 (5) for atoms Cl1 and Cl2. The dihedral angle formed by the benzene rings is 4.57 (11)°. The molecular conformation is stabilized by an intramolecular O—H···N hydrogen bond (Table 1). In the crystal packing, the molecules are linked via intermolecular C—H···O hydrogen bonds into zig-zag chains running parallel to the *b* axis. In addition, intermolecular Br···Cl and Cl···Cl interactions are observed (Fig. 2) falling in the typical range of halogen-halogen interactions (Saruma & Desiraju, 1986, Moorthy *et al.*,  2002): Br1···Cl1^i^ = 3.5289 (11) Å; Cl1···Cl2^ii^ = 3.5042 (12) Å; symmetry codes: (i) x, 3/2-y, -1/2+z; (ii) 1+x, y, 1+z.

### Experimental

3-Bromo-5-chlorosalicylaldehyde(0.1 mmol, 23.6 mg) and 2-chlorobenzenamine (0.1 mmol, 12.8 mg) were dissolved in methanol (10 ml). The mixture was stirred at room temperature for 1 h and then filtered. After allowing the filtrate to stand in air for 7 d, yellow block-shaped crystals of the title compound were formed by slow evaporation of the solvent. The crystals were collected, washed with methanol and dried in a vacuum desiccator using anhydrous CaCl_2_ (yield 52%).

### Refinement

All H atoms were placed in geometrically idealized positions and constrained to ride on their parent atoms, with C—H = 0.93 Å, O—H = 0.82 Å and *U*_iso_(H) = = 1.2 *U*_eq_(C, O).

### Crystal data

**Table:**

C_13_H_8_BrCl_2_NO	*F*(000) = 680
*M**_r_* = 345.01	*D*_x_ = 1.755 Mg m^−^^3^
Monoclinic, *P*2_1_/*c*	Mo *K*α radiation, λ = 0.71073 Å
Hall symbol: -P 2ybc	Cell parameters from 2353 reflections
*a* = 8.4299 (10) Å	θ = 2.4–25.2°
*b* = 14.0115 (16) Å	µ = 3.54 mm^−^^1^
*c* = 11.4194 (14) Å	*T* = 298 K
β = 104.512 (1)°	Block, yellow
*V* = 1305.8 (3) Å^3^	0.45 × 0.38 × 0.36 mm
*Z* = 4	

### Data collection

**Table:**

Siemens SMART CCD area-detector diffractometer	2295 independent reflections
Radiation source: fine-focus sealed tube	1726 reflections with *I* > 2σ(*I*)
graphite	*R*_int_ = 0.038
φ and ω scans	θ_max_ = 25.0°, θ_min_ = 2.4°
Absorption correction: multi-scan (*SADABS*; Siemens, 1996)	*h* = −10→9
*T*_min_ = 0.230, *T*_max_ = 0.279	*k* = −14→16
6450 measured reflections	*l* = −13→13

### Refinement

**Table:**

Refinement on *F*^2^	Secondary atom site location: difference Fourier map
Least-squares matrix: full	Hydrogen site location: inferred from neighbouring sites
*R*[*F*^2^ > 2σ(*F*^2^)] = 0.032	H-atom parameters constrained
*wR*(*F*^2^) = 0.077	*w* = 1/[σ^2^(*F*_o_^2^) + (0.0256*P*)^2^ + 0.9771*P*] where *P* = (*F*_o_^2^ + 2*F*_c_^2^)/3
*S* = 1.03	(Δ/σ)_max_ = 0.001
2295 reflections	Δρ_max_ = 0.39 e Å^−^^3^
164 parameters	Δρ_min_ = −0.57 e Å^−^^3^
0 restraints	Extinction correction: *SHELXL97* (Sheldrick, 2008), Fc^*^=kFc[1+0.001xFc^2^λ^3^/sin(2θ)]^-1/4^
Primary atom site location: structure-invariant direct methods	Extinction coefficient: 0.0127 (8)

### Special details

**Table:**

Geometry. All e.s.d.'s (except the e.s.d. in the dihedral angle between two l.s. planes) are estimated using the full covariance matrix. The cell e.s.d.'s are taken into account individually in the estimation of e.s.d.'s in distances, angles and torsion angles; correlations between e.s.d.'s in cell parameters are only used when they are defined by crystal symmetry. An approximate (isotropic) treatment of cell e.s.d.'s is used for estimating e.s.d.'s involving l.s. planes.
Refinement. Refinement of *F*^2^ against ALL reflections. The weighted *R*-factor *wR* and goodness of fit *S* are based on *F*^2^, conventional *R*-factors *R* are based on *F*, with *F* set to zero for negative *F*^2^. The threshold expression of *F*^2^ > σ(*F*^2^) is used only for calculating *R*-factors(gt) *etc*. and is not relevant to the choice of reflections for refinement. *R*-factors based on *F*^2^ are statistically about twice as large as those based on *F*, and *R*- factors based on ALL data will be even larger.

### Fractional atomic coordinates and isotropic or equivalent isotropic displacement parameters (Å^2^)

**Table:**

	*x*	*y*	*z*	*U*_iso_*/*U*_eq_	
Br1	0.54350 (5)	0.75675 (2)	0.54203 (4)	0.06096 (18)	
Cl1	0.70198 (11)	0.53128 (7)	0.95100 (8)	0.0596 (3)	
Cl2	−0.01427 (12)	0.49417 (8)	0.22925 (8)	0.0767 (3)	
N1	0.1581 (3)	0.43674 (18)	0.4723 (2)	0.0434 (7)	
O1	0.3040 (3)	0.59685 (15)	0.45663 (19)	0.0500 (6)	
H1	0.2386	0.5532	0.4344	0.075*	
C1	0.2481 (4)	0.4266 (2)	0.5798 (3)	0.0436 (8)	
H1A	0.2359	0.3725	0.6238	0.052*	
C2	0.3687 (4)	0.4978 (2)	0.6346 (3)	0.0381 (7)	
C3	0.3916 (4)	0.5800 (2)	0.5697 (3)	0.0387 (7)	
C4	0.5108 (4)	0.6456 (2)	0.6259 (3)	0.0420 (8)	
C5	0.6058 (4)	0.6305 (2)	0.7418 (3)	0.0461 (8)	
H5	0.6860	0.6744	0.7777	0.055*	
C6	0.5809 (4)	0.5499 (2)	0.8042 (3)	0.0453 (8)	
C7	0.4644 (4)	0.4838 (2)	0.7520 (3)	0.0436 (8)	
H7	0.4492	0.4296	0.7950	0.052*	
C8	0.0439 (4)	0.3675 (2)	0.4143 (3)	0.0457 (8)	
C9	−0.0435 (4)	0.3866 (3)	0.2963 (3)	0.0528 (9)	
C10	−0.1548 (5)	0.3222 (4)	0.2313 (4)	0.0741 (13)	
H10	−0.2122	0.3362	0.1524	0.089*	
C11	−0.1804 (5)	0.2369 (3)	0.2839 (5)	0.0814 (14)	
H11	−0.2550	0.1931	0.2403	0.098*	
C12	−0.0967 (5)	0.2165 (3)	0.3997 (5)	0.0751 (12)	
H12	−0.1145	0.1589	0.4349	0.090*	
C13	0.0149 (5)	0.2818 (3)	0.4650 (4)	0.0622 (10)	
H13	0.0711	0.2677	0.5440	0.075*	

### Atomic displacement parameters (Å^2^)

**Table:**

	*U*^11^	*U*^22^	*U*^33^	*U*^12^	*U*^13^	*U*^23^
Br1	0.0721 (3)	0.0385 (2)	0.0755 (3)	−0.00233 (18)	0.0246 (2)	0.00929 (18)
Cl1	0.0696 (6)	0.0611 (6)	0.0377 (5)	0.0104 (5)	−0.0059 (4)	−0.0064 (4)
Cl2	0.0649 (6)	0.1111 (9)	0.0489 (6)	−0.0086 (6)	0.0043 (5)	0.0204 (6)
N1	0.0443 (15)	0.0433 (15)	0.0400 (16)	−0.0012 (12)	0.0058 (13)	−0.0021 (13)
O1	0.0568 (14)	0.0463 (13)	0.0419 (14)	−0.0019 (11)	0.0033 (11)	0.0102 (11)
C1	0.053 (2)	0.0336 (17)	0.045 (2)	0.0022 (15)	0.0139 (17)	0.0005 (15)
C2	0.0456 (18)	0.0347 (16)	0.0339 (16)	0.0025 (14)	0.0100 (14)	−0.0008 (14)
C3	0.0413 (18)	0.0372 (17)	0.0376 (18)	0.0075 (14)	0.0100 (15)	0.0012 (14)
C4	0.0472 (19)	0.0302 (16)	0.051 (2)	0.0046 (14)	0.0159 (16)	0.0037 (14)
C5	0.0457 (19)	0.0396 (18)	0.051 (2)	0.0001 (15)	0.0078 (17)	−0.0093 (16)
C6	0.052 (2)	0.0444 (19)	0.0365 (18)	0.0114 (16)	0.0050 (15)	−0.0022 (15)
C7	0.056 (2)	0.0365 (17)	0.0375 (18)	0.0046 (16)	0.0096 (16)	0.0017 (15)
C8	0.0420 (18)	0.048 (2)	0.047 (2)	0.0008 (15)	0.0108 (16)	−0.0116 (16)
C9	0.042 (2)	0.073 (2)	0.043 (2)	0.0002 (18)	0.0108 (16)	−0.0120 (18)
C10	0.057 (2)	0.111 (4)	0.052 (2)	−0.013 (2)	0.011 (2)	−0.029 (3)
C11	0.066 (3)	0.088 (3)	0.091 (4)	−0.021 (2)	0.020 (3)	−0.052 (3)
C12	0.073 (3)	0.055 (2)	0.099 (4)	−0.018 (2)	0.023 (3)	−0.022 (3)
C13	0.065 (2)	0.051 (2)	0.065 (3)	−0.0089 (19)	0.006 (2)	−0.0048 (19)

### Geometric parameters (Å, °)

**Table:**

Br1—C4	1.885 (3)	C5—H5	0.9300
Cl1—C6	1.750 (3)	C6—C7	1.373 (5)
Cl2—C9	1.736 (4)	C7—H7	0.9300
N1—C1	1.279 (4)	C8—C13	1.381 (5)
N1—C8	1.409 (4)	C8—C9	1.390 (5)
O1—C3	1.338 (3)	C9—C10	1.377 (5)
O1—H1	0.8200	C10—C11	1.379 (6)
C1—C2	1.449 (4)	C10—H10	0.9300
C1—H1A	0.9300	C11—C12	1.364 (7)
C2—C7	1.394 (4)	C11—H11	0.9300
C2—C3	1.409 (4)	C12—C13	1.388 (5)
C3—C4	1.394 (4)	C12—H12	0.9300
C4—C5	1.381 (4)	C13—H13	0.9300
C5—C6	1.378 (4)		
			
C1—N1—C8	123.1 (3)	C6—C7—H7	120.0
C3—O1—H1	109.5	C2—C7—H7	120.0
N1—C1—C2	121.4 (3)	C13—C8—C9	117.8 (3)
N1—C1—H1A	119.3	C13—C8—N1	125.0 (3)
C2—C1—H1A	119.3	C9—C8—N1	117.2 (3)
C7—C2—C3	119.9 (3)	C10—C9—C8	121.4 (4)
C7—C2—C1	119.5 (3)	C10—C9—Cl2	118.9 (3)
C3—C2—C1	120.6 (3)	C8—C9—Cl2	119.7 (3)
O1—C3—C4	119.2 (3)	C9—C10—C11	119.5 (4)
O1—C3—C2	122.4 (3)	C9—C10—H10	120.3
C4—C3—C2	118.3 (3)	C11—C10—H10	120.3
C5—C4—C3	121.2 (3)	C12—C11—C10	120.3 (4)
C5—C4—Br1	119.4 (2)	C12—C11—H11	119.8
C3—C4—Br1	119.4 (2)	C10—C11—H11	119.8
C6—C5—C4	119.6 (3)	C11—C12—C13	119.9 (4)
C6—C5—H5	120.2	C11—C12—H12	120.1
C4—C5—H5	120.2	C13—C12—H12	120.1
C7—C6—C5	120.9 (3)	C8—C13—C12	121.1 (4)
C7—C6—Cl1	119.8 (3)	C8—C13—H13	119.5
C5—C6—Cl1	119.3 (3)	C12—C13—H13	119.5
C6—C7—C2	120.1 (3)		
			
C8—N1—C1—C2	177.5 (3)	Cl1—C6—C7—C2	−179.4 (2)
N1—C1—C2—C7	−179.9 (3)	C3—C2—C7—C6	−0.2 (5)
N1—C1—C2—C3	−0.8 (5)	C1—C2—C7—C6	178.9 (3)
C7—C2—C3—O1	179.6 (3)	C1—N1—C8—C13	0.1 (5)
C1—C2—C3—O1	0.6 (4)	C1—N1—C8—C9	−178.6 (3)
C7—C2—C3—C4	0.0 (4)	C13—C8—C9—C10	−0.4 (5)
C1—C2—C3—C4	−179.1 (3)	N1—C8—C9—C10	178.4 (3)
O1—C3—C4—C5	−179.1 (3)	C13—C8—C9—Cl2	178.9 (3)
C2—C3—C4—C5	0.6 (4)	N1—C8—C9—Cl2	−2.3 (4)
O1—C3—C4—Br1	0.8 (4)	C8—C9—C10—C11	0.0 (6)
C2—C3—C4—Br1	−179.6 (2)	Cl2—C9—C10—C11	−179.3 (3)
C3—C4—C5—C6	−1.0 (5)	C9—C10—C11—C12	0.3 (6)
Br1—C4—C5—C6	179.2 (2)	C10—C11—C12—C13	−0.1 (7)
C4—C5—C6—C7	0.8 (5)	C9—C8—C13—C12	0.6 (6)
C4—C5—C6—Cl1	180.0 (2)	N1—C8—C13—C12	−178.1 (3)
C5—C6—C7—C2	−0.2 (5)	C11—C12—C13—C8	−0.4 (6)

### Hydrogen-bond geometry (Å, °)

**Table:**

*D*—H···*A*	*D*—H	H···*A*	*D*···*A*	*D*—H···*A*
O1—H1···N1	0.82	1.86	2.586 (3)	147
C11—H11···O1^i^	0.93	2.56	3.324 (5)	139

Symmetry codes: (i) −*x*, *y*−1/2, −*z*+1/2.
